# Control of Insulin Secretion by Production of Reactive Oxygen Species: Study Performed in Pancreatic Islets from Fed and 48-Hour Fasted Wistar Rats

**DOI:** 10.1371/journal.pone.0158166

**Published:** 2016-06-30

**Authors:** Ana Cláudia Munhoz, Patrícia Riva, Daniel Simões, Rui Curi, Angelo Rafael Carpinelli

**Affiliations:** Department of Physiology and Biophysics, Institute of Biomedical Sciences, University of São Paulo, São Paulo, Brazil; INSERM UMRS 1138, FRANCE

## Abstract

Mitochondria and NADPH oxidase are important sources of reactive oxygen species in particular the superoxide radical (ROS) in pancreatic islets. These molecules derived from molecular oxygen are involved in pancreatic β-cells signaling and control of insulin secretion. We examined the involvement of ROS produced through NADPH oxidase in the leucine- and/or glucose-induced insulin secretion by pancreatic islets from fed or 48-hour fasted rats. Glucose-stimulated insulin secretion (GSIS) in isolated islets was evaluated at low (2.8 mM) or high (16.7 mM) glucose concentrations in the presence or absence of leucine (20 mM) and/or NADPH oxidase inhibitors (VAS2870–20 μM or diphenylene iodonium—DPI—5 μM). ROS production was determined in islets treated with dihydroethidium (DHE) or MitoSOX Red reagent for 20 min and dispersed for fluorescence measurement by flow cytometry. NADPH content variation was examined in INS-1E cells (an insulin secreting cell line) after incubation in the presence of glucose (2.8 or 16.7 mM) and leucine (20 mM). At 2.8 mM glucose, VAS2870 and DPI reduced net ROS production (by 30%) and increased GSIS (by 70%) in a negative correlation manner (r = -0.93). At 16.7 mM glucose or 20 mM leucine, both NADPH oxidase inhibitors did not alter insulin secretion neither net ROS production. Pentose phosphate pathway inhibition by treatment with DHEA (75 μM) at low glucose led to an increase in net ROS production in pancreatic islets from fed rats (by 40%) and induced a marked increase (by 144%) in islets from 48-hour fasted rats. The NADPH/NADP^+^ ratio was increased when INS-1E cells were exposed to high glucose (by 4.3-fold) or leucine (by 3-fold). In conclusion, increased ROS production through NADPH oxidase prevents the occurrence of hypoglycemia in fasting conditions, however, in the presence of high glucose or high leucine levels, the increased production of NADPH and the consequent enhancement of the activity of the antioxidant defenses mitigate the excess of ROS production and allow the secretory process of insulin to take place.

## Introduction

Our group has shown [1] that isolated rat pancreatic islets express a neutrophil-like nicotinamide adenine dinucleotide phosphate oxidase (NADPH oxidase), an enzyme complex that forms superoxide (^•^O_2_^-^) utilizing NADPH as electron donor [2, 3]. This enzyme complex is an important source of superoxide during the process of insulin secretion induced by glucose (GSIS), interleukins (e.g. IL-1β) or fatty acids (e.g palmitic acid, oleic acid, linoleic acid and γ-linolenic acid) [4–10]. Alteration in the NADPH oxidase activity does impair the process of GSIS by insulin secreting cell lines and pancreatic islets obtained from mice and rats [3–7]. Cytosolic superoxide dismutase (SOD1) converts ^•^O_2_^-^ into hydrogen peroxide (H_2_O_2_) [11]. The H_2_O_2_ formed is removed by the action of other antioxidant enzymes (such as glutathione peroxidase-GPx), which activity is dependent on NADPH mainly produced through the pentose-phosphate pathway (PPP) [12–15]. Decreases in reactive oxygen species (ROS) content by scavenging system occurs with increase in glucose utilization by pancreatic β-cells in a dose and time-dependent manner [14]. Hydrogen peroxide inhibits glucose decarboxylation and insulin secretion by isolated rat pancreatic islets as we previously reported [16]. In fact, isolated islets show high net production of superoxide radical when exposed to low glucose concentration (2.8 mM).

The involvement of superoxide radical in insulin release under low and high glucose levels *in vivo* remains unclarified. During fasting, pancreatic islets are exposed to low blood glucose concentration that affects β-cell metabolism and insulin secretion and content. GSIS is impaired in pancreas isolated from rodents after fasting for 16 [17], 24 [18, 19], 48 [17, 19–24], 72 [19, 25, 26], 96 ([24, 27], and 192 hours [27]. Changes in pancreatic islet secretory machinery (e.g. decrease in pancreatic content of mRNAs for insulin and GLUT-2) [28] are involved in the impairment of GSIS induced by fasting [22–24, 29]. The 48-hour period exhibits all marked changes reported on insulin secretion and so it is not necessary to submit the animal to longer periods of fasting. On the other hand, shorter periods may jeopardize the fasting consequences due to the gastrointestinal transit and coprophagy.

In the present study, pancreatic islets from rats submitted to 48-hour fasting were used to examine the control of insulin secretion by ROS, in particular superoxide radical, in a more physiological way. We evaluated productions of ROS and insulin secretion in pancreatic islets isolated from fed and 48-hour fasted rats. The islet incubation was performed in the presence of glucose (2.8 or 16.7 mM), leucine (20 mM), NADPH oxidase inhibitors (3-benzyl-7-(2-benzoxazolyl)thio-1,2,3-triazolo(4,5-d)pyrimidine—VAS2870, 20 μM; and diphenylene iodonium—DPI, 5 μM), and an inhibitor of the pentose-phosphate pathway (dehydroepiandrosterone—DHEA). Changes in the NADPH/NADP^+^ ratio associated with ROS production were evaluated in INS-1E cells cultivated in the presence of glucose (2.8 or 16.7 mM) and leucine (20 mM).

## Material and Methods

### Ethical approval

Ethical Committee on Animal Research of the Institute of Biomedical Sciences of the University of São Paulo (CEUA) and Brazilian Society of Science in Laboratory Animals (SBCAL) approved the experimental protocols of this study including that involved in the use of 48-hour fasted rats. The approved protocol number is 080 as stated in the sheet 130 of the book 02.

### Experimental protocols

#### Protocol 1

Pancreatic islets from fed rats were incubated for 120 minutes in the presence of 2.8 or 16.7 mM glucose. Kinetic cytosolic ROS production was measured every 5 minutes.

#### Protocol 2

Pancreatic islets from fed rats were incubated for 120 minutes in the presence of 2.8 mM glucose or for 60 minutes with 2.8 mM glucose that was replaced by 16.7 mM glucose and then maintained for another 60-minute period incubation. Kinetic cytosolic ROS production was measured every 5 minutes.

#### Protocol 3

Pancreatic islets from fed rats were incubated for 60 minutes in the presence of 2.8 or 16.7 mM glucose with or without addition of leucine (20 mM). Cytosolic ROS production was measured at the end of the incubation period.

#### Protocol 4

Pancreatic islets from fed or 48-hour fasted rats were incubated for 60 minutes in the presence of 2.8 mM glucose with or without addition of VAS2870 (20 μM) or DPI (5 μM), associated or not with leucine (20 mM). Cytosolic and mitochondrial ROS production and insulin secretion were measured at the end of the incubation period.

#### Protocol 5

Pancreatic islets from fed or 48-hour fasted rats were incubated for 60 minutes in the presence of 2.8 mM glucose with or without addition of DHEA (75 μM). Cytosolic ROS production was measured at the end of the incubation period.

#### Protocol 6

INS-1E cells were incubated for 60 minutes in the presence of 2.8 or 16.7 mM glucose with or without addition of leucine (20 mM). NADPH/NADP^+^ ratio was measured at the end of the incubation period.

### Animals

Female Wistar rats aged 2–3 months with 250 ± 20 gram body weight were housed in cages with five animals each in a room with constant temperature of 23 ± 2° Celsius on a 12-hour light/dark cycle. Fed rats had free access to water and standard diet (Nuvilab, São Paulo, SP, Brazil) whereas fasted rats had free access to water but were submitted to food deprivation from 09:00 a.m. until to the time of the experiments after 48 hours. Preliminary experiments showed marked effects of fasting on pancreatic islets only after 48 hours.

### Isolation of pancreatic islets

Isolation of pancreatic islets was carried out by the method of exocrine pancreas digestion using collagenase [30]. The rats were euthanized and a midline laparotomy was performed. The bile duct was clamped at its distal end where a needle was introduced to inject 20 mL of collagenase solution (0.68 mg/mL—Sigma-Aldrich, St. Louis, MO, USA). The pancreas was dissected, placed in a bath at 37°C for 25 minutes and shaken by hand for 1 minute for exocrine pancreas digestion. The sample was washed three times to remove exocrine pancreas and pancreatic islets were collected in a Petri dish using a micropipette and a stereoscope. Similar procedure was used in our previous studies [8, 31–33].

### Static insulin secretion

Groups of 5 islets isolated from fed or fasted Wistar rats were placed in a microtube and pre-incubated for 30 minutes in Krebs-Henseleit buffer containing 0.1% albumin and 5.6 mM glucose and then incubated for 1 hour at 37°C with 2.8 or 16.7 mM glucose associated or not with 20 mM leucine, VAS2870 (20 μM) or DPI (5 μM). After incubation, 300 μL of the supernatant were stored at -20°C for determination of secreted insulin. The islets were disrupted for measurement of intracellular insulin content. The amounts of secreted and intracellular insulin were determined by radioimmunoassay (RIA) [34].

### Measurement of net cytosolic ROS production

Groups of 20 islets isolated from fed or fasted Wistar rats were placed in a microtube and pre-incubated for 30 minutes in Krebs-Henseleit buffer containing 5.6 mM glucose and 0.1% albumin, followed by 1 hour incubation at 37°C with: a) 2.8 or 16.7 mM glucose associated or not with 20 mM leucine; b) 2.8 mM glucose associated or not with 20 mM leucine, VAS2870 (20 μM) or DPI (5 μM); c) 2.8 mM glucose associated or not with the inhibitor of the pentose-phosphate pathway dehydroepiandrosterone (DHEA-75 μM). Afterwards, the samples were kept at room temperature for 20 minutes with 50 μM dihydroethidium (DHE– Life Technologies, Eugene, Oregon, EUA) [35].

The incubation solution was removed and islets were treated with 300 μL trypsin for 2 minutes at 37°C. Afterwards, 600 μL RPMI-1640 culture medium were added to the tubes for trypsin inactivation. The supernatant was discarded and the islets homogenized in 200 μL RPMI-1640 culture medium for complete cell dispersion. The samples were placed in a 96 well plate and analyzed by flow cytometry (Guava EasyCyte 8HT—Millipore, Billerica, MA, USA).

DHE is a redox sensitive probe that has been used to label live cells and to measure production of ROS in particular superoxide. DHE is oxidized by reactive oxygen and nitrogen species (e.g. superoxide, hydrogen peroxide, hydroxyl radical, and peroxynitrite) and forms ethidium. DHE oxidation by superoxide only generates 2-hydroxyethidium. Both eithidium and 2-hydroxyethidium bind to DNA and stain the cells in a cumulative way (excitation 535 nm, emission 610 nm) [36–40] despite the short half-life of superoxide radical. Therefore, we measured herein the net production of ROS in particular superoxide.

### Measurement of net mitochondrial ROS production

The same protocol described above was used, except that MitoSOX Red reagent (Life Technologies, Eugene, Oregon, USA—5 μM) substituted DHE. MitoSOX Red reagent is a fluorogenic dye ethidium-based probe specifically targeted to mitochondria. MitoSOX was developed by conjugating methyltriphenylphosphonium ion, a lipophilic cation that accumulates in energized mitochondria [41], to DHE. The accumulation of red fluorescence (excitation 510 nm, emission 580 nm) indicates the net production of ROS in the mitochondria as described above for cytosol [42–46].

### Kinetic measurement of net cytosolic ROS production

Groups of 20 islets isolated from Wistar rats were pre-incubated for 30 minutes in 500 μL Krebs-Henseleit buffer containing 5.6 mM glucose and 0.1% bovine serum albumin. Islets were treated with 100 μL trypsin at 37°C for 3 minutes, inactivated with 100 μL RPMI 1640 culture medium, transferred to 1.5 mL tubes and centrifuged during 3 minutes at room temperature. The supernatant was discarded and the islets homogenized in 200 μL RPMI-1640 culture medium for complete cell dispersion. The samples were then transferred to 96 well plates with 200 μL Krebs-Henseleit buffer containing DHE (50 μM) and: a) 2.8 mM or 16.7 mM glucose for 120 minutes; b) 2.8 or 2.8 replaced by 16.7 mM glucose after 60 minutes and maintained for another period of 60 minutes. Fluorescence was read each 5-minute period (Multi-Detection Microplate Reader—BioTek) during 120 minutes.

### Measurement of the NADPH/NADP^+^ ratio in INS-1E cells

The measurement of NADPH was not performed in isolated islets since it would require too many islets and consequently too many animals. The measurement of the NADPH/NADP^+^ ratio was then carried out in INS-1E cells by using the NADPH/NADP^+^ Quantification Kit (Sigma-Aldrich, St. Louis, MO, USA). The INS-1E insulin secreting cells were pre-incubated for 30 minutes in Krebs-Henseleit-buffer containing 0.1% albumin and 5.6 mM glucose and then incubated for 1 hour at 37°C with 2.8 or 16.7 mM glucose associated or not with 20 mM leucine. After incubation, the supernatant was discarded and 150 μL extraction buffer were added. Sonicated and deproteinized samples were then placed in a 96 well plate along with 100 μL NADP cycling buffer and NADP cycling enzyme mix, and were incubated for 5 minutes at room temperature. Ten μL of NADPH developer were added to each well and after 2 hours, the samples were analyzed by fluorometry (Synergy^™^ H1 Hybrid Multi-Mode Microplate Reader—BioTek).

### Statistical analysis

Results are expressed as mean ± standard error of the mean. Student’s t-test or analysis of variance (ANOVA) followed by Dunnett´s post-test was used when appropriate as indicated. Comparisons were considered as significantly different for p<0.05. The GraphPad Prism 5 software was used for the analysis.

## Results

### Glucose control of net ROS production by pancreatic islets from fed rats

**Fig 1:**
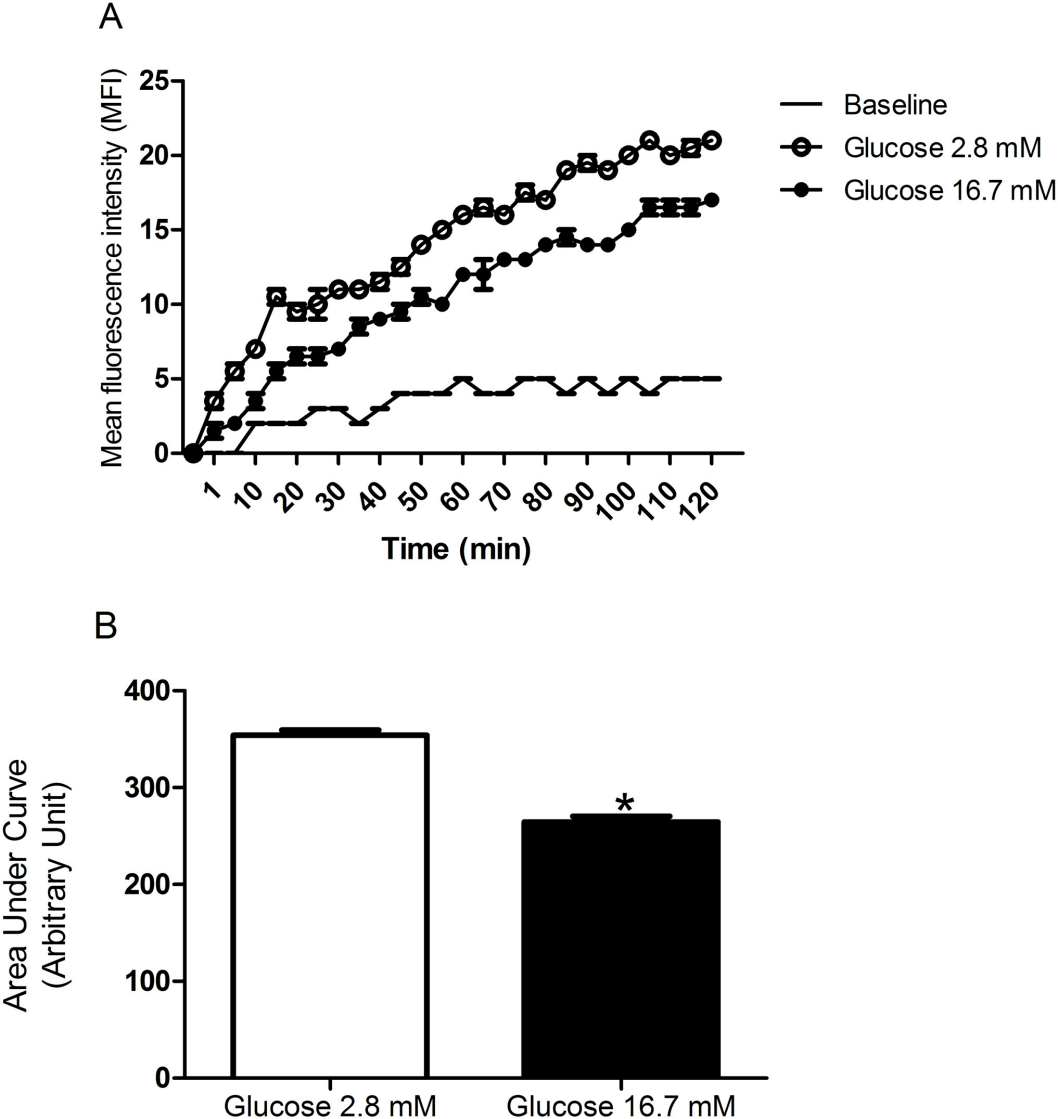
Kinetic measurement of net cytosolic ROS production. **(A)** Mean fluorescence intensity (MFI—arbitrary units) by pancreatic islets isolated from fed rats, treated with dihydroethidium (DHE), and measured every 5 minutes during 120-minute incubation in the presence of 2.8 or 16.7 mM glucose—experimental protocol 1. **(B)** The corresponding areas under the curves were then calculated. The results are presented as mean ± SEM of four cell preparations for each group. * p <0.05 as compared to 2.8 mM glucose and indicated by the Student’s t-test.

**Fig 2:**
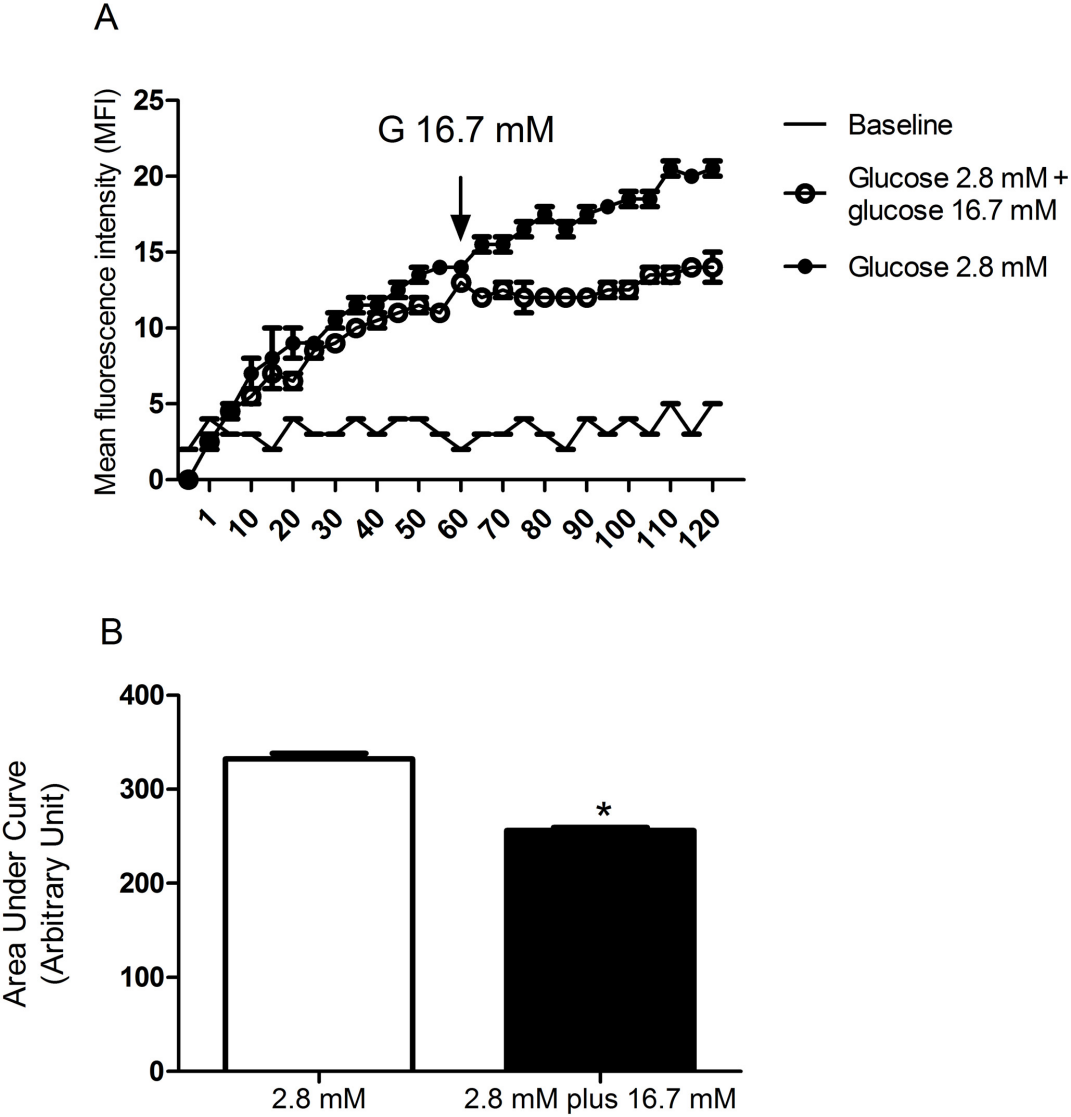
Kinetic measurement of net cytosolic ROS production. **(A)** Mean fluorescence intensity (MFI—arbitrary units) by pancreatic islets isolated from fed rats, treated with dihydroethidium (DHE), and incubated with 2.8 mM glucose for 120 minutes or for 60 minutes with 2.8 mM glucose that was replaced by 16.7 mM glucose and then maintained for another 60-minute period incubation—experimental protocol 2. **(B)** The corresponding areas under the curves were then calculated. The results are presented as mean ± SEM of five cell preparations for each group. *p <0.05 as compared to 2.8 mM glucose and indicated by the Student’s t-test.

ROS production by pancreatic islets was significantly higher after 120-minute incubation in the presence of 2.8 mM as compared to 16.7 mM glucose (Fig 1A and 1B), as indicated by the lower area under the curve (33%) at high glucose level. The replacement of 2.8 mM by 16.7 mM glucose in the medium led to a significant decrease in net ROS production by pancreatic islets (Fig 2A and 2B) as indicated by a significant lower area under the curve (29%) at high glucose level.

### Net ROS production by pancreatic islets from fed rats in the presence of 20 mM leucine and 2.8 or 16.7 mM glucose

**Fig 3:**
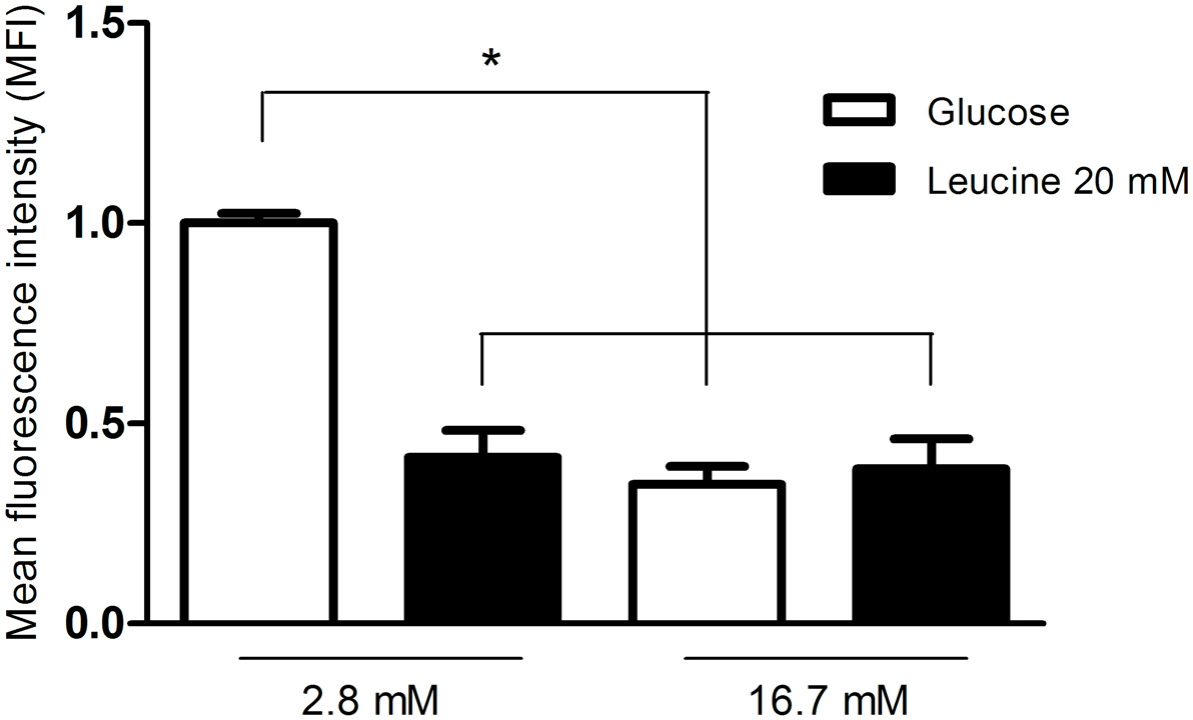
Net ROS production by pancreatic islets from fed rats in the presence of 20 mM leucine and 2.8 or 16.7 mM glucose. Mean fluorescence intensity (MFI—arbitrary units) by pancreatic islets from fed rats, treated with dihydroethidium (DHE), and incubated for 60 minutes in the presence of 2.8 or 16.7 mM glucose with or without addition of leucine (LEU—20 mM)–experimental protocol 3. The results are presented as mean ± SEM of four cell preparations for each group. *p <0.05 as compared to 2.8 mM glucose only and indicated by one-way ANOVA and Dunnett's post test.

Net ROS production by pancreatic islets was significantly lower after one-hour incubation in the presence of 20 mM leucine with 2.8 mM glucose as compared to 2.8 mM glucose only (Fig 3). However, in the presence of 16.7 mM glucose, leucine (20 mM) did not change net ROS production in comparison with 16.7 mM glucose only.

### ROS production and insulin secretion by pancreatic islets from fed and fasted rats in the presence of 2.8 mM glucose with 20 mM leucine and NADPH oxidase inhibitors

**Fig 4:**
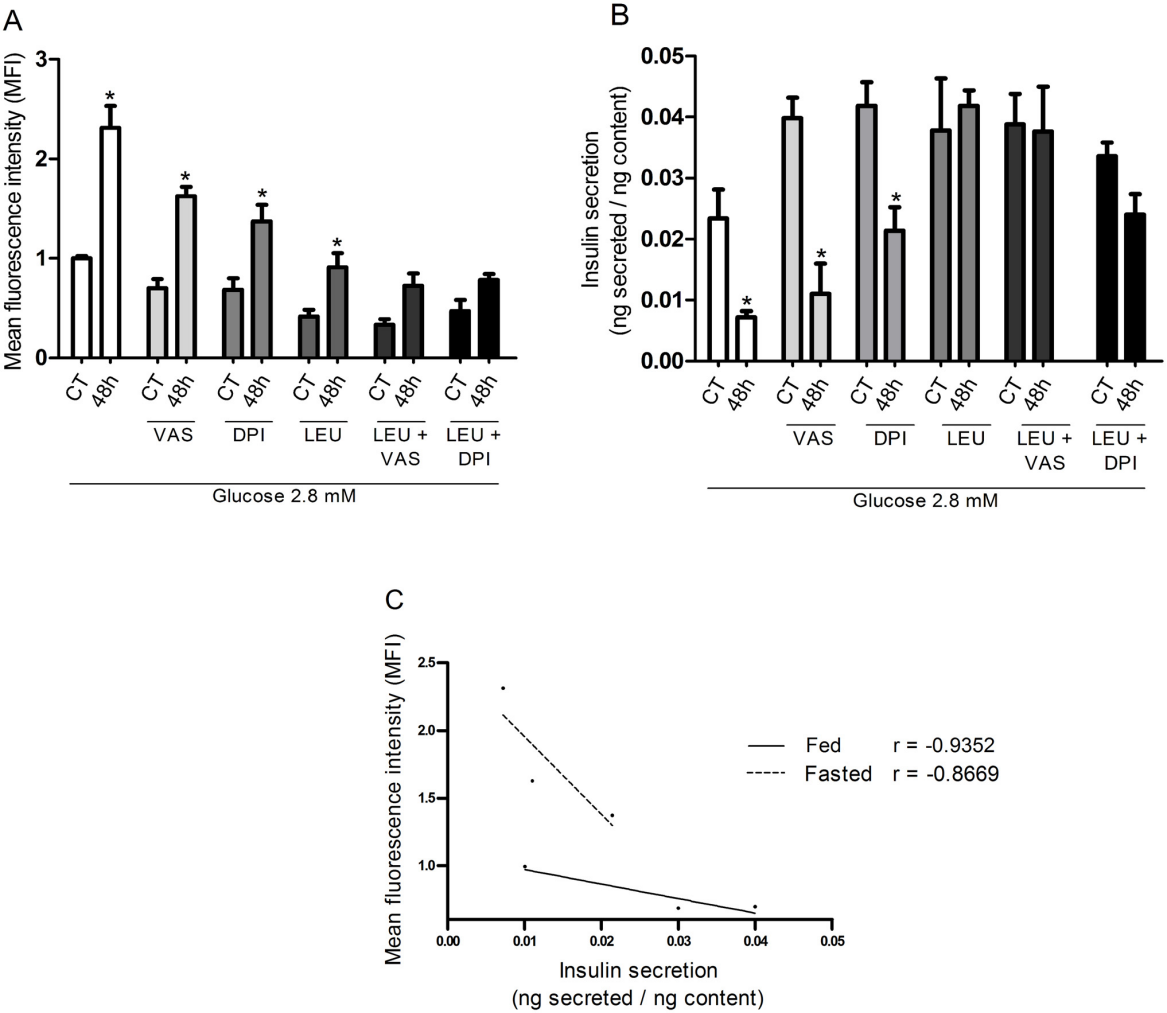
ROS production and insulin secretion by pancreatic islets from fed and fasted rats in the presence of 20 mM leucine and 2.8 or 16.7 mM glucose. **(A)** Mean fluorescence intensity (MFI—arbitrary units) and **(B)** insulin secretion by pancreatic islets from fed (CT) or 48-hour fasted (48h) rats, treated with dihydroethidium (DHE), and incubated for 60 minutes in the presence of 2.8 mM glucose with or without addition of VAS2870 (20 μM) or DPI (5 μM), associated or not with leucine (LEU—20 mM)–experimental protocol 4. The results are presented as mean ± SEM of four different cell preparations for each group * p <0.05 compared to fed control (CT) under the same conditions as indicated by the Student t-test. **(C)** Inverse correlation between MFI and insulin secretion in the presence of 2.8 mM glucose with or without addition of VAS2870 (20 μM) or DPI (5 μM) in pancreatic islets isolated from fed or 48-hour fasted rats as indicated by the correlation test using the GraphPad Prism 5.

Net ROS production in the presence of 2.8 mM glucose by pancreatic islets obtained from 48-hour fasted rats was significantly higher (by 123%) compared with those from fed rats (Fig 4A). Insulin secretion in the presence of 2.8 mM glucose by isolated islets obtained from 48-hour fasted rats was significantly lower (by 69%) compared with fed rats. This effect did not occur in the presence of 20 mM leucine (Fig 4B) despite the decrease in net ROS production. The additions of VAS and DPI reduced net ROS production and caused an increase of insulin secretion in pancreatic islets isolated from 48-hour fasted rats. The inverse correlation between insulin secretion and net production of ROS under the treatments with VAS and DPI is indicated in the graphs. Correlation, however, was not observed when pancreatic islets were treated with 2.8 mM glucose plus 20 mM leucine. Under this latter condition, insulin secretion was enhanced as compared to 2.8 mM glucose only but net ROS production remained unchanged.

Treatment with both NADPH oxidase pharmacological inhibitors caused a significant decrease in net ROS production at 2.8 mM glucose and an increase of insulin secretion by pancreatic islets obtained from fed rats in an inverse (r = −0.93) correlation manner. The same was observed (r = -0.86) in pancreatic islets obtained from fasted rats (Fig 4C).

### Changes in ROS production by pentose phosphate pathway inhibition

**Fig 5:**
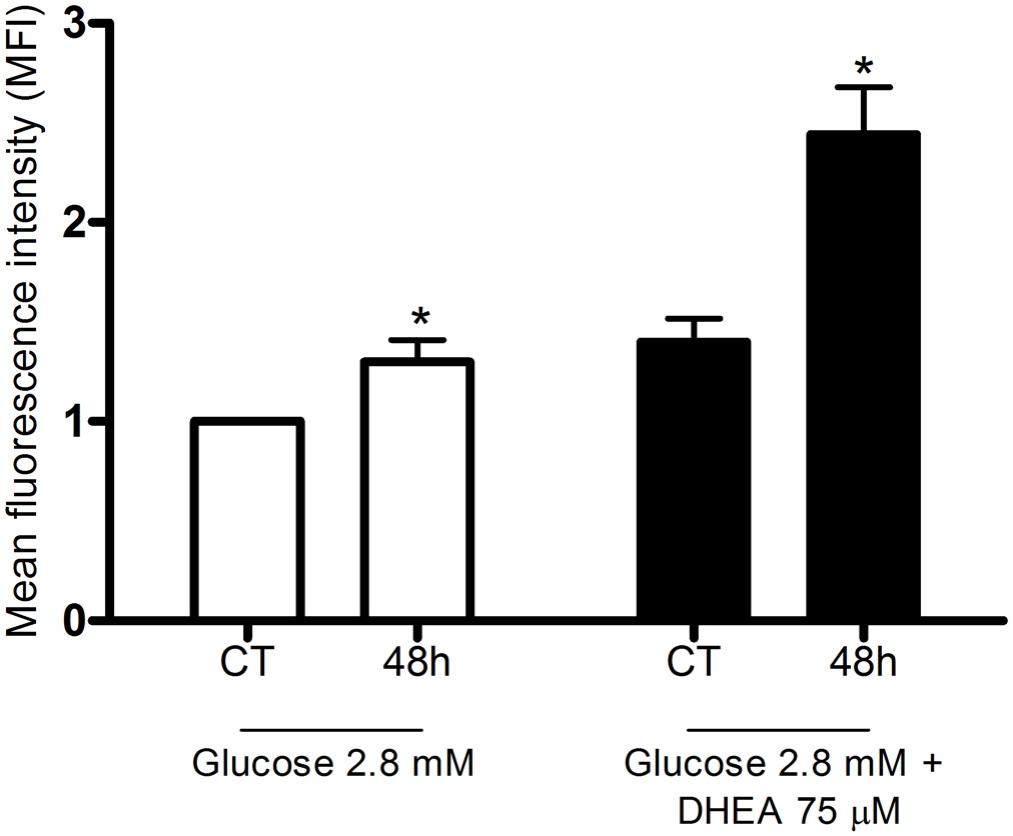
Changes in ROS production induced by pentose phosphate pathway inhibition. Mean fluorescence intensity (MFI—arbitrary units) by pancreatic islets from fed (CT) or 48-hour fasted (48h) rats, treated with dihydroethidium (DHE), and incubated for 60 minutes in the presence of 2.8 mM glucose with or without addition of dehydroepiandrosterone (DHEA—75 μM)–experimental protocol 5. The results are presented as mean ± SEM of five different cell preparations for each group. * p <0.05 compared to fed control (CT) under the same conditions as indicated by the Student t-test.

Increasing concentrations of dehydroepiandrosterone (DHEA), an inhibitor of the pentose phosphate pathway (PPP), raised net ROS production by pancreatic islets from fed rats in the presence of 2.8 mM glucose. Islets isolated from 48-hour fasted rats, however, showed increased net production of ROS and the treatment with DHEA (75 μM) caused an additional increment (Fig 5).

### Changes of NADPH/NADP+ ratio in INS-1E cells cultivated in the presence of 20 mM leucine and 2.8 or 16.7 mM glucose

**Fig 6:**
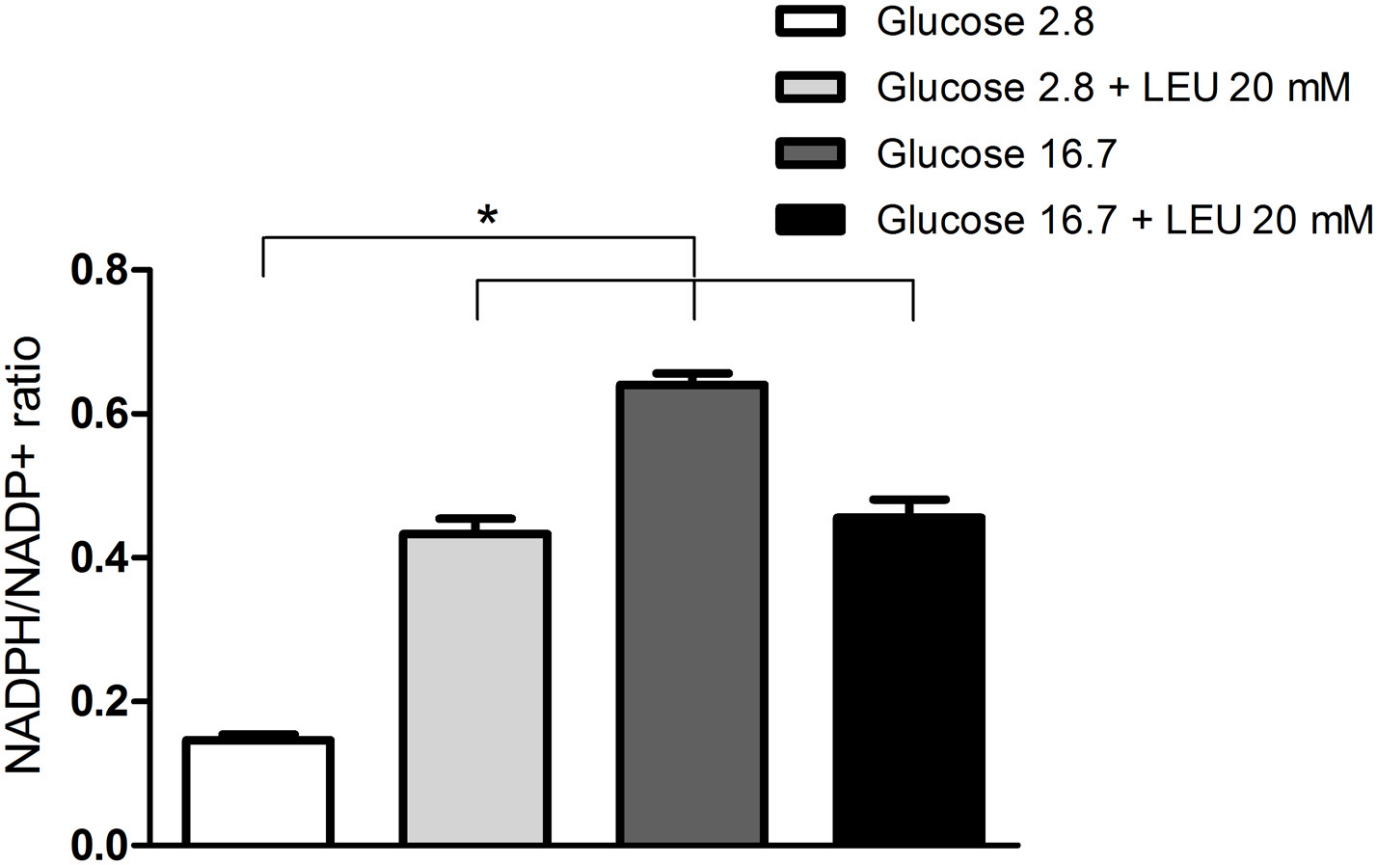
Changes of NADPH/NADP+ ratio in INS-1E cells cultivated in the presence of 20 mM leucine and 2.8 or 16.7 mM glucose. NADPH/NADP^+^ ratio in INS-1E cells incubated for 60 minutes in the presence of 2.8 or 16.7 mM glucose with or without addition of leucine (LEU—20 mM)–experimental protocol 6. The results are presented as mean ± SEM of three different cell preparations for each group. *p <0.05 compared to 2.8 mM glucose as indicated by the Student t-test.

The NADPH/NADP^+^ ratio was increased after treatment with leucine or higher glucose concentration (16.7 mM) when compared to 2.8 mM glucose (Fig 6).

### Mitochondrial ROS measurement

**Fig 7:**
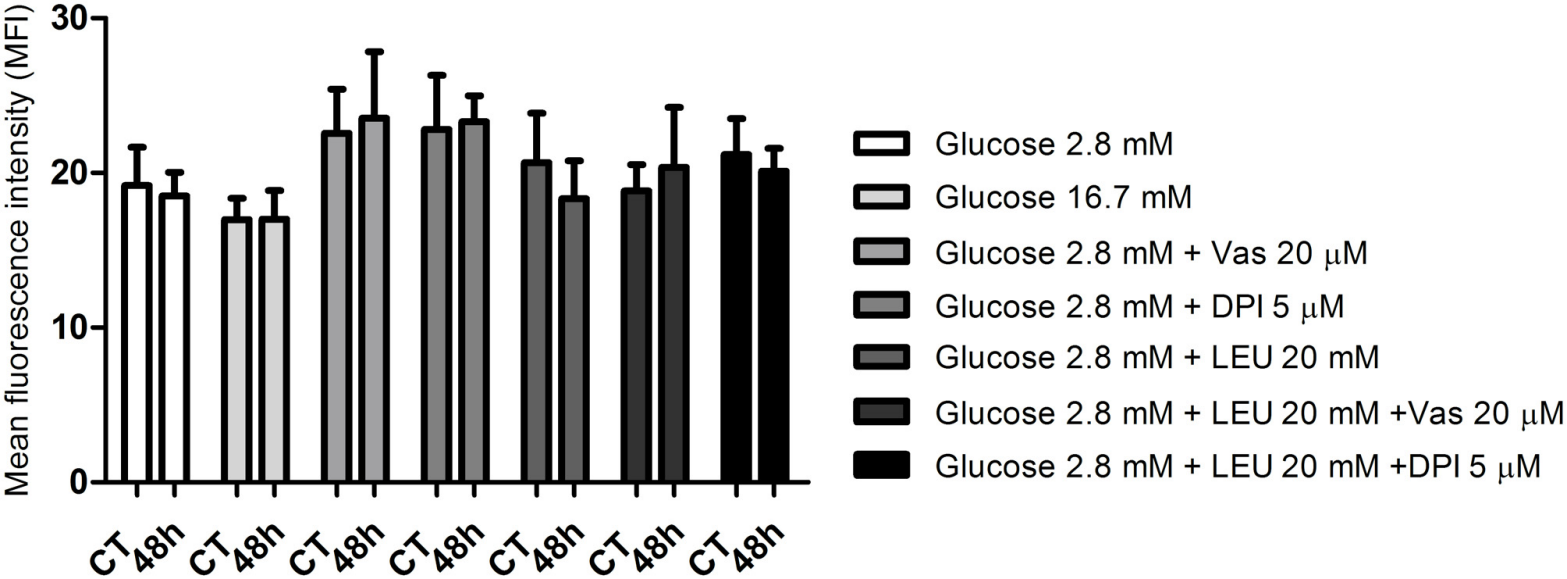
Mitochondrial ROS measurement. Mean fluorescence intensity (MFI—arbitrary units) by pancreatic islets from fed (CT) or 48-hour fasted (48h) rats. The islets were treated with MitoSOX Red reagent and incubated for 60 minutes in the presence of 2.8 or 16.7 mM glucose with or without addition of VAS2870 (20 μM) or DPI (5 μM) associated or not with leucine (LEU—20 mM)—experimental protocol 4. The results are presented as mean ± SEM of four cell preparations for each group as indicated by one-way ANOVA and Dunnett's post test.

There was no significant difference in net mitochondrial ROS production by isolated islets obtained from fed or 48-hour fasted rats in all conditions studied (Fig 7).

## Discussion

The increases in glucose concentration reduced net ROS production (Figs 1–3) by pancreatic islets. These findings confirm previous reports glucose loading decreases net production of ^•^O_2_^-^ [47] and H_2_O_2_ parallel with an increase in NADPH concentrations in insulin secreting cells [4, 16, 48–50]. Pancreatic β-cells show low antioxidant enzyme activities compared to other tissues, in particular glutathione peroxidase (GPx), the only enzyme present in the cytosol, which can inactivate H_2_O_2_ generated through SOD1 action [51]. The maintenance of the redox state at high glucose concentration is achieved mainly through activation of the pentose phosphate pathway (PPP) and production of NADPH [12, 13, 49, 50]. Despite low antioxidant defense activity, pancreatic β-cell machinery can handle with increased production of oxidant molecules through an efficient production of NADPH in healthy conditions. This does not apply to conditions associated to unbalanced antioxidant enzyme activities that results in oxidative stress [52].

Our group [14] showed that DHEA, an inhibitor of glucose-6-phosphate dehydrogenase (G6PDH), the rate-limiting enzyme of the PPP [53], leads to an increase in net ROS production and markedly decreases glucose-stimulated insulin secretion. The DHEA effect is abolished by treatments with antioxidant agents such as N-acetyl-L-cysteine (NAC) and catalase (PEG-CAT) [14]. The protective effect of NAC on MIN6 pancreatic tumor cell lines abolishing oxidative stress and apoptosis induced by aldosterone has been reported [54]. NAC provides cysteine for the formation of reduced glutathione (GSH) increasing the GSH/GSSG ratio and so antioxidant cell defense systems. Diabetes-associated clinical features such as glucose intolerance, loss of pancreatic β-cell mass and increased oxidative stress are then attenuated [54].

**Fig 8:**
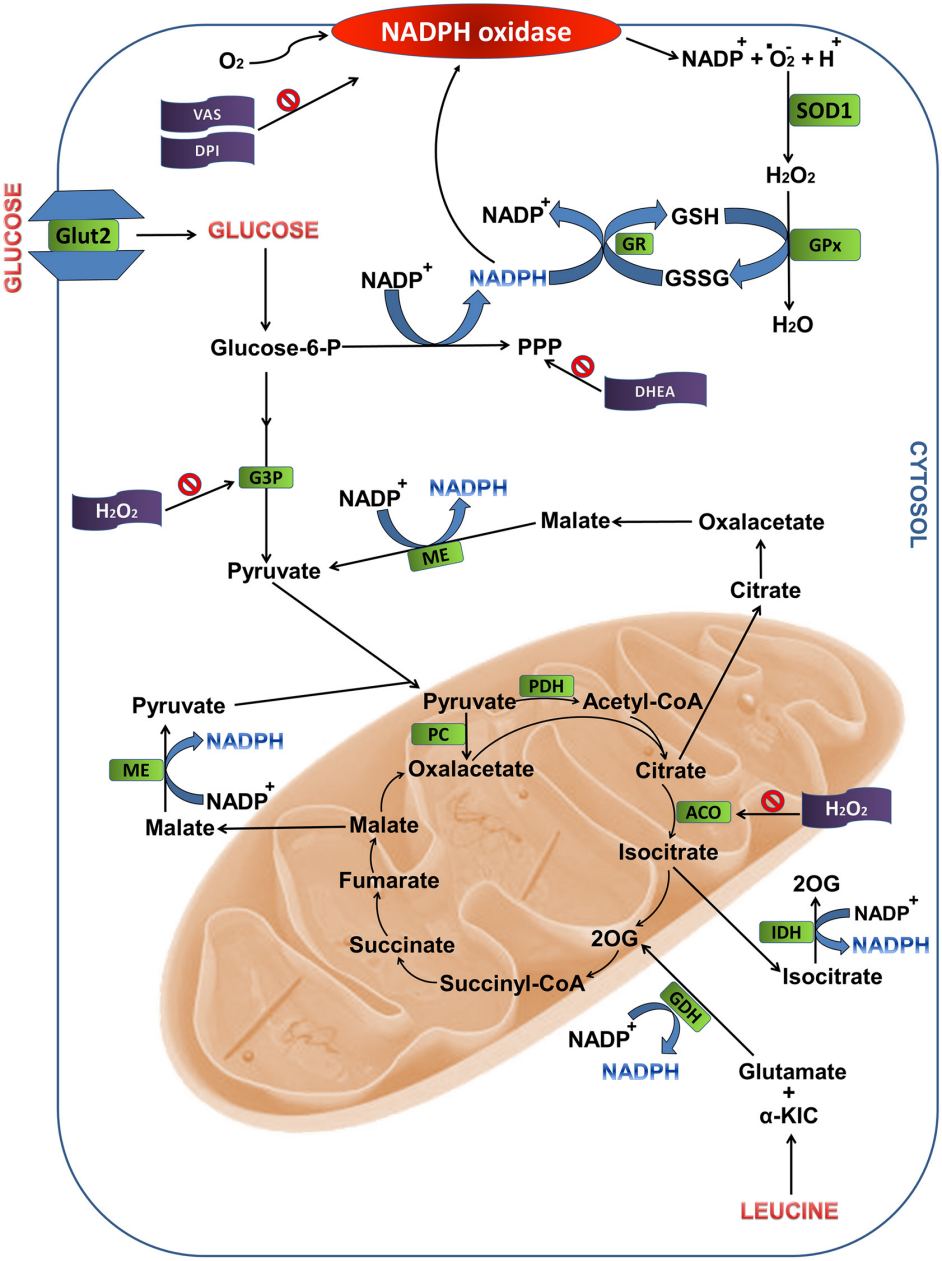
Metabolic pathways associated to NADPH production from glucose and leucine oxidation in pancreatic islets. The sites indicated are: the pentose phosphate pathway and reactions involving intermediates of the Krebs cycle. ACO, aconitase; CYT, cytosol; DHEA, dehydroepiandrosterone; G3P, glyceraldehyde-3-phosphate dehydrogenase; GDH, glutamate dehydrogenase; GPx, glutathione peroxidase; GR, glutathione reductase; GSH, reduced glutathione; GSSG, oxidized glutathione; IDH, isocitrate dehydrogenase; ME, malic enzyme; MIT, mitochondria; PDH, pyruvate dehydrogenase; PPP, pentose phosphate pathway; SOD1, superoxide dismutase 1; 2OG, 2-oxoglutarate; α-KIC, α-ketoisocaproic acid.

DHEA (75 μM) in the presence of 2.8 mM glucose caused a 44% increase in net ROS production by pancreatic islets obtained from 48-hour fasted rats compared with islets obtained from fed rats (Fig 5). These results confirm the observation that activation of the PPP promotes a decrease of net ROS production by pancreatic β-cells possibly by supplying NADPH for reduced glutathione formation [49]. Although NADPH oxidase uses NADPH to generate ^•^O_2_^-^ [50] (Fig 8), in the presence of high glucose levels this nucleotide is deviated to production of antioxidant molecules that remove ROS and prevent oxidative stress [49]. In addition to NADPH oxidase, mitochondria also generate superoxide radical as byproducts of mitochondrial electron transport chain. Islets from fasted rats showed similar net ROS production in mitochondria compared with those from fed rats (Fig 7).

The low net ROS production by pancreatic islets in the presence of 2.8 mM glucose plus 20 mM leucine as compared to 2.8 mM glucose only (Fig 3) can be explained by metabolism of leucine through the Krebs cycle. Leucine leads to an increase in citrate production that in association with ATP inhibits phosphofructokinase activity, the most important regulatory enzymes in the glycolytic pathway that catalyzes the conversion of fructose 6-phosphate and ATP to fructose 1,6-bisphosphate and ADP [55, 56]. The increase in acetyl-CoA formation, also as a product of leucine metabolism, inhibits pyruvate dehydrogenase activity, resulting in accumulation of pyruvate. These metabolic changes promote inhibition of glycolysis facilitating the deviation of the flux of substrates from glycolysis to the PPP and so the increase in NADPH production [49].

Furthermore, leucine metabolism also leads to formation of α-ketoisocaproic acid (α-KIC) and glutamate. α-KIC enters the Krebs cycle and is metabolized to acetyl-CoA. Glutamate is deaminated by glutamate dehydrogenase forming 2-oxoglutarate with subsequent formation of NADPH (Fig 8). Leucine metabolism leads to an increase in 2-oxoglutarate and intermediates of the citric acid cycle, such as citrate, which, in turn, when in high concentration, migrates to cytosol through translocase tricarboxylate, where it is converted to oxaloacetate and acetyl-CoA by citrate lyase. Oxaloacetate is converted to malate that in turn is oxidized to pyruvate by malic enzyme forming NADPH. Citrate is converted to isocitrate that through the cytosolic isocitrate dehydrogenase also generates NADPH (Fig 8).

The decrease in net ROS production (about 30%) through NADPH oxidase inhibition in the presence of VAS2870 or DPI by pancreatic islets exposed to 2.8 mM glucose (Fig 4) confirms the participation of this enzyme complex in the maintenance of the pancreatic β-cells redox state [2, 57]. The effects of NADPH oxidase inhibitors and leucine reported for 2.8 mM glucose were not observed at 16.7 mM glucose (S8 Fig). This latter observation was probably due to optimally activated state of the antioxidant defense systems in high glucose levels and/or leucine metabolism as described above.

Another ROS H_2_O_2_ has been shown to inhibit glyceraldehyde-3-phosphate dehydrogenase (glycolytic pathway) and aconitase (Krebs cycle) activities and insulin secretion [14–16, 58, 59]. Herein, an inverse correlation (Fig 4C) was found between the decrease in the net ROS production induced by the inhibitors of NADPH oxidase and the elevation in insulin secretion at 2.8 mM glucose.

The net production of ROS was increased whereas insulin secretion was decreased in pancreatic islets from 48-hour fasted as compared to fed rats. This is an *in vivo* evidence for the suppressing effect of ROS on insulin secretion reported *in vitro* [14]. In fact, the fasting condition pronounced the inverse correlation between the mean fluorescence intensity (MFI) and insulin secretion observed in pancreatic islets from fed animals (Fig 4C). Possible circulating factors associated with the changes observed in insulin secretion and oxidative stress in pancreatic β-cells from 48-hour fasted rats remain to be investigated.

VAS2870 and DPI also decreased net ROS production by pancreatic islets in a negative correlation manner with insulin secretion being an additional evidence for the participation of NADPH oxidase and radical superoxide in this secretory process (Fig 4). In spite of the fact that leucine, at 2.8 mM glucose, decreased net production of ROS by pancreatic islets, insulin secretion was raised under this condition. This effect was probably due to elevation of ATP/ADP ratio as a consequence of leucine metabolism through the Krebs cycle (Fig 8).

## Conclusions

The present work shows that increased ROS in particular superoxide radical production through NADPH oxidase may contribute to avoid the occurrence of hypoglycemia in fasting conditions.

## Supporting Information

S1 Fig Net ROS production by pancreatic islets from fed rats in the presence of 2.8 or 16.7 mM glucose associated with different concentrations of VAS2870. (JPG)

S2 Fig Net ROS production by pancreatic islets from fed rats in the presence of 2.8 or 16.7 mM glucose associated with different concentrations of DPI. (JPG)

S3 Fig Net ROS production by pancreatic islets from fed rats in the presence of 2.8 or 16.7 mM glucose associated with different concentrations of DHEA. (JPG)

S4 Fig Net ROS production by pancreatic islets from fed or 24 and 48-hour fasted rats in the presence of 2.8 mM glucose associated with DHEA 75 μM, VAS 20 μM, DPI 5 μM and leucine 20 mM. (JPG)

S5 Fig Net ROS production by pancreatic islets from fed or 24 and 48-hour fasted rats in the presence of 16.7 mM glucose associated with DHEA 75 μM, VAS 20 μM, DPI 5 μM and leucine 20 mM. (JPG)

S6 Fig Net ROS production by pancreatic islets from fed rats in the presence of 2.8 or 16.7 mM glucose associated with DHEA 75 μM and NAC 100 μM. (JPG)

S7 Fig Cell viability by pancreatic islets from fed rats in the presence of 2.8 or 16.7 mM glucose associated with DHEA 75 μM and NAC 100 μM. (JPG)

S8 Fig Net ROS production by pancreatic islets from fed or 24 and 48-hour fasted rats in the presence of 16.7 mM glucose associated with VAS 20 μM, DPI 5 μM and leucine 20 mM. (JPG)
