# Supplementary figures and images for: Control of Insulin Secretion by Production of Reactive Oxygen Species: Study Performed in Pancreatic Islets from Fed and 48-Hour Fasted Wistar Rats
Source: PLoS One. 2016 Jun 30;11(6):e0158166. doi: 10.1371/journal.pone.0158166 (PMC4928816; doi:10.1371/journal.pone.0158166)

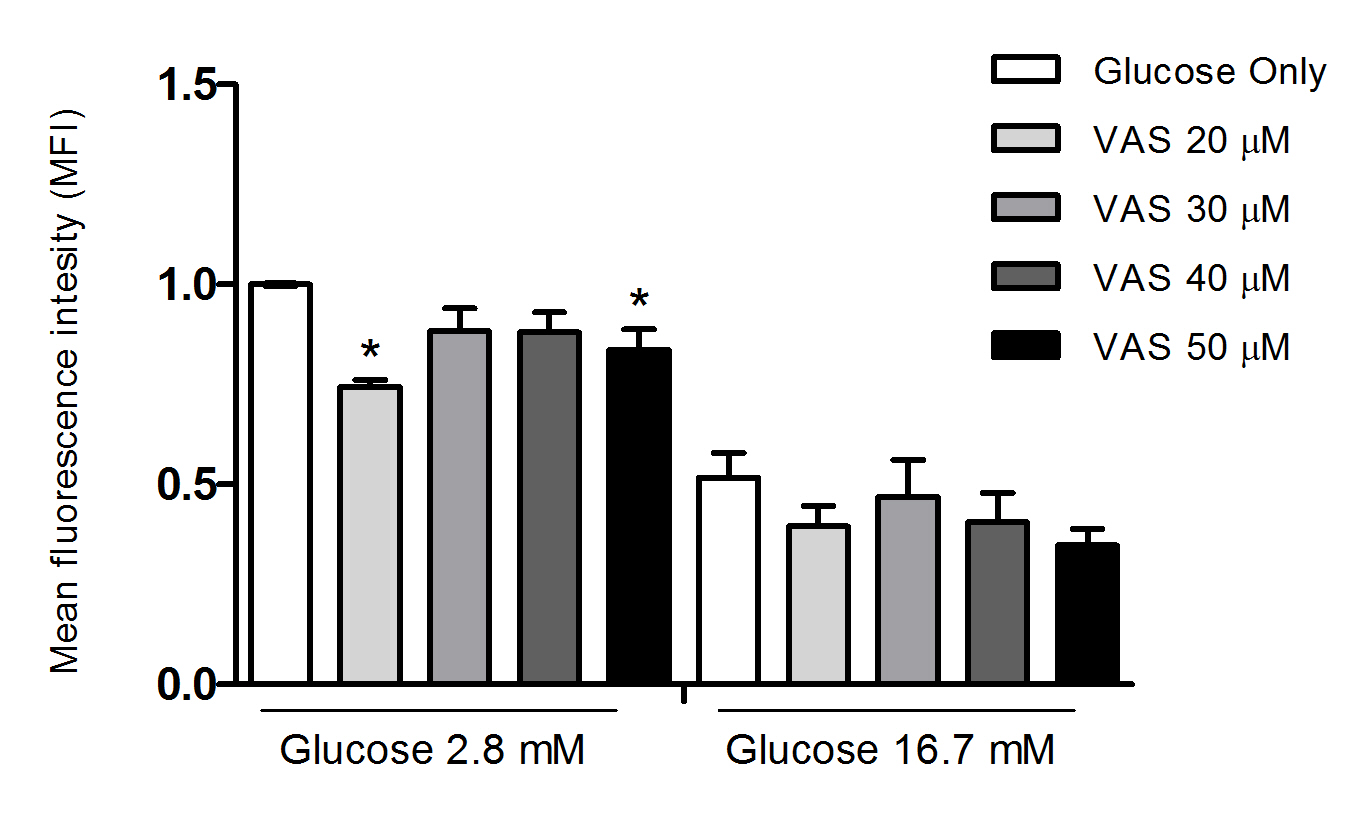

Supplement: S1 Fig — (JPG) [file pone.0158166.s001.jpg]

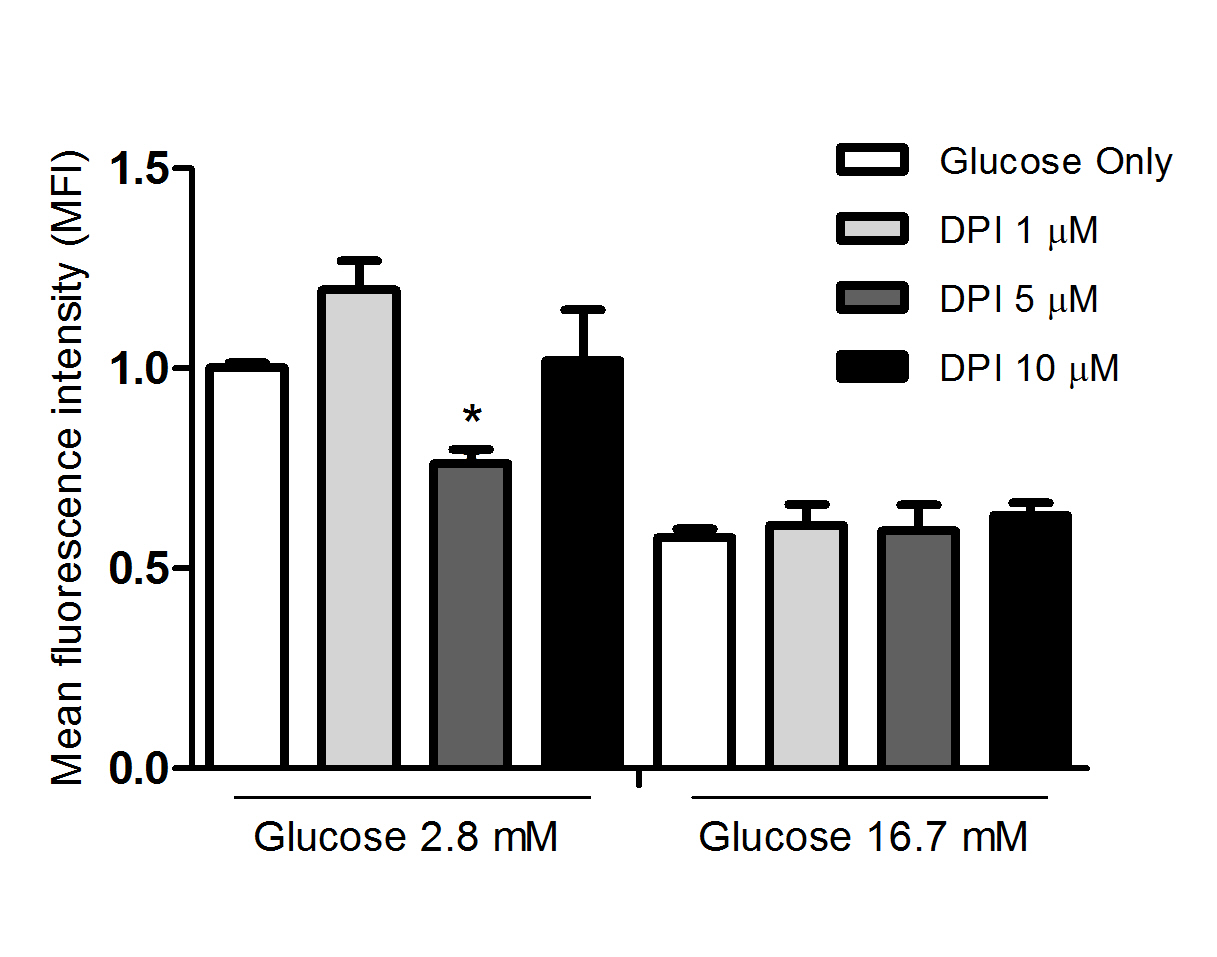

Supplement: S2 Fig — (JPG) [file pone.0158166.s002.jpg]

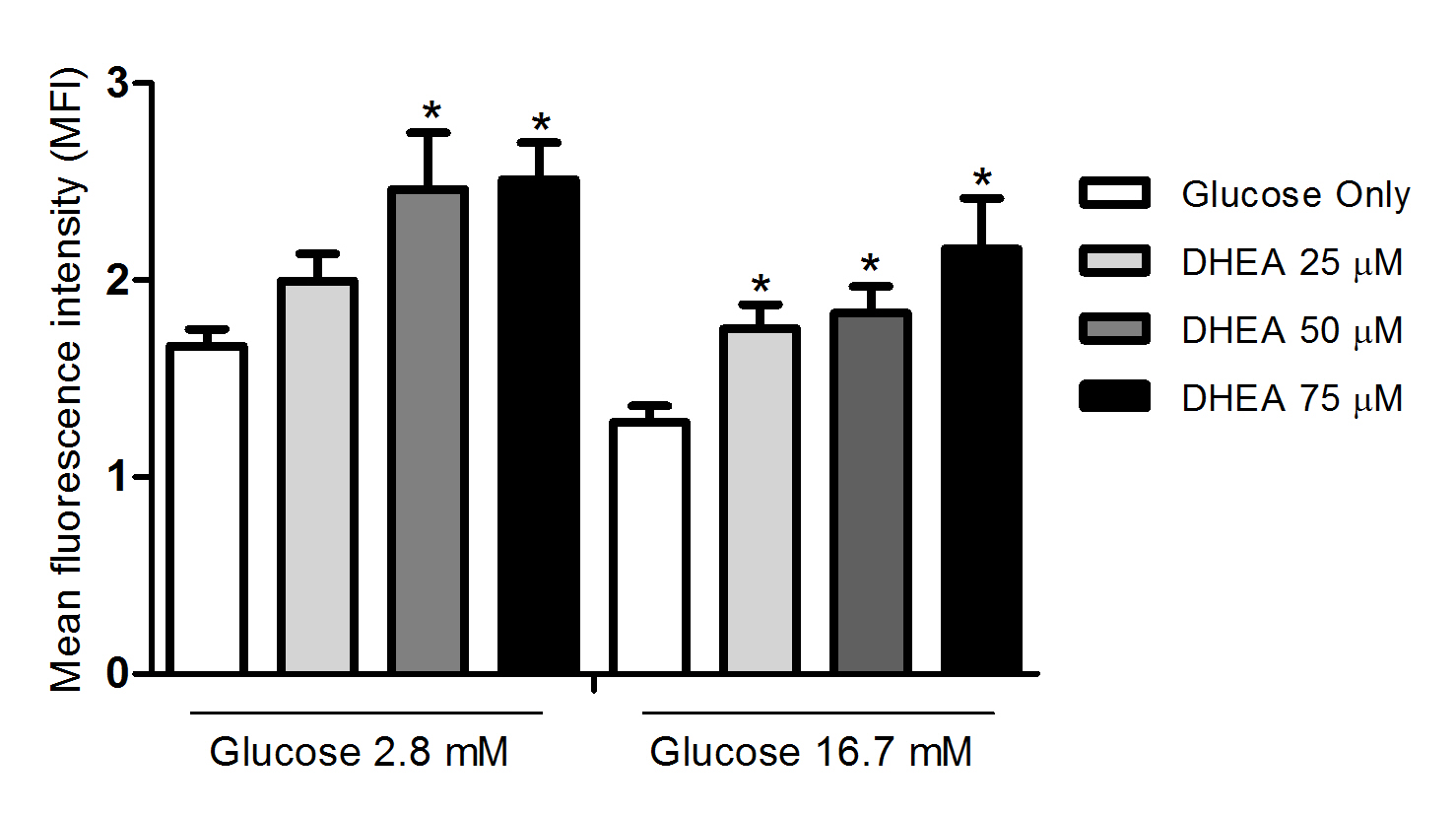

Supplement: S3 Fig — (JPG) [file pone.0158166.s003.jpg]

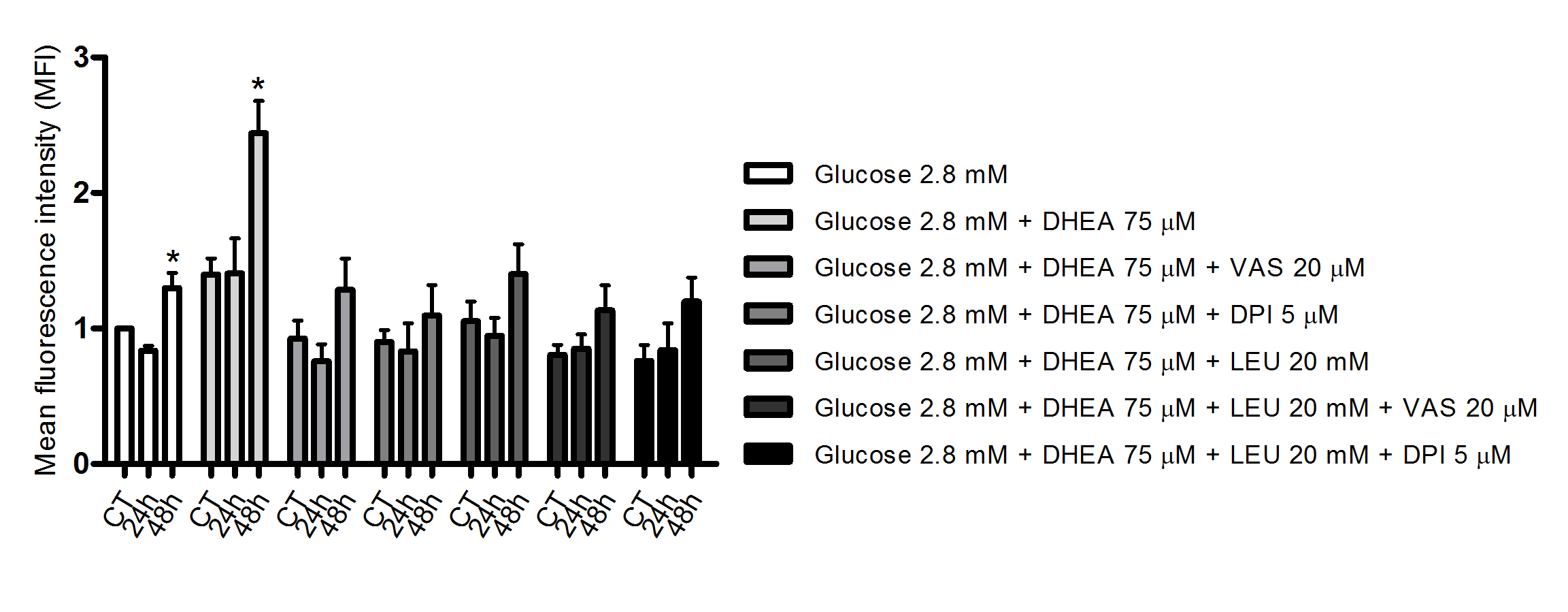

Supplement: S4 Fig — (JPG) [file pone.0158166.s004.jpg]

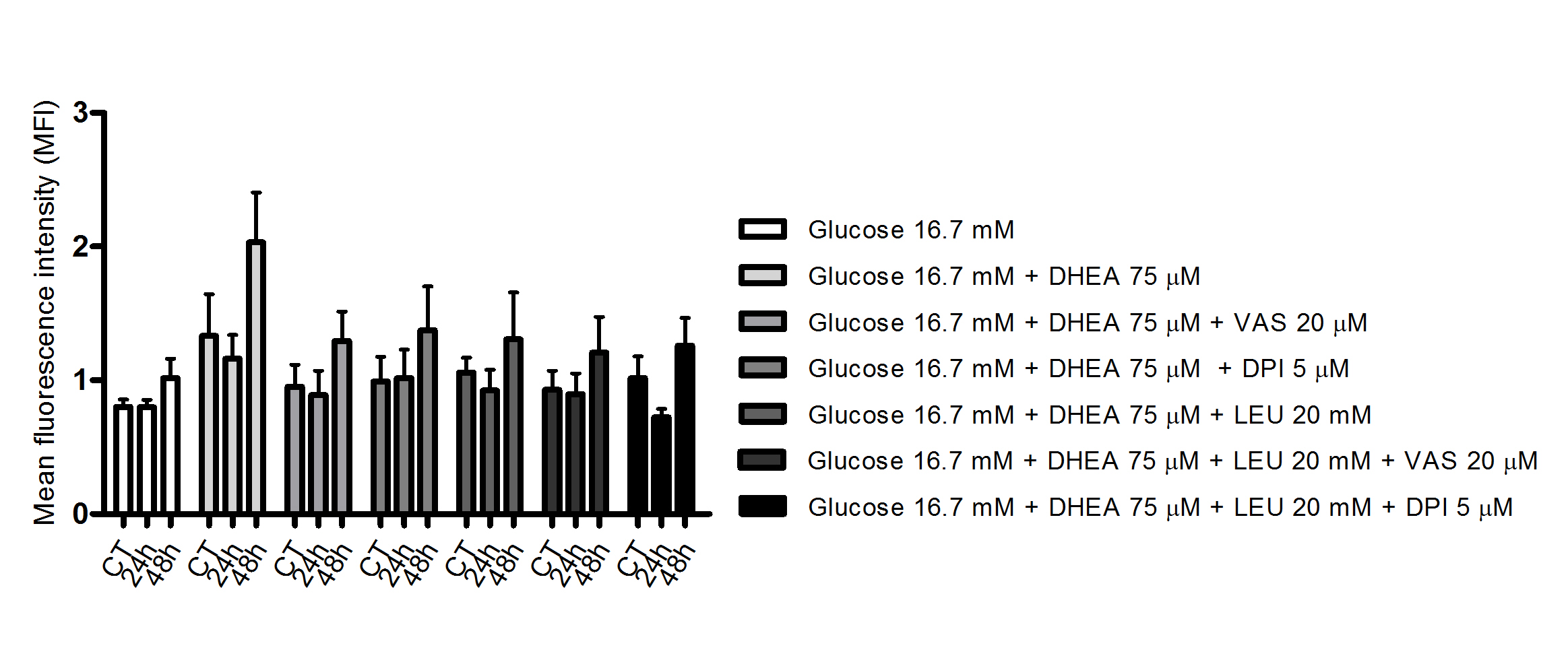

Supplement: S5 Fig — (JPG) [file pone.0158166.s005.jpg]

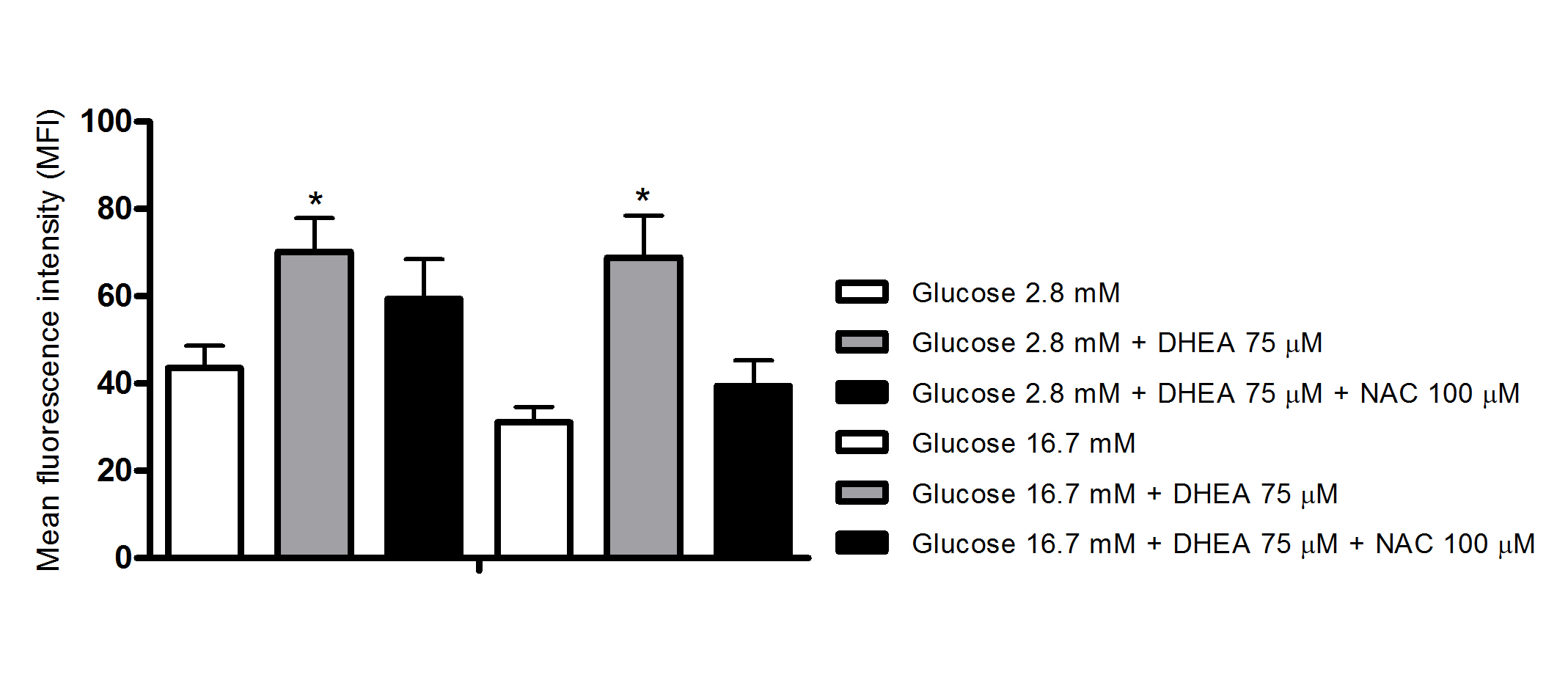

Supplement: S6 Fig — (JPG) [file pone.0158166.s006.jpg]

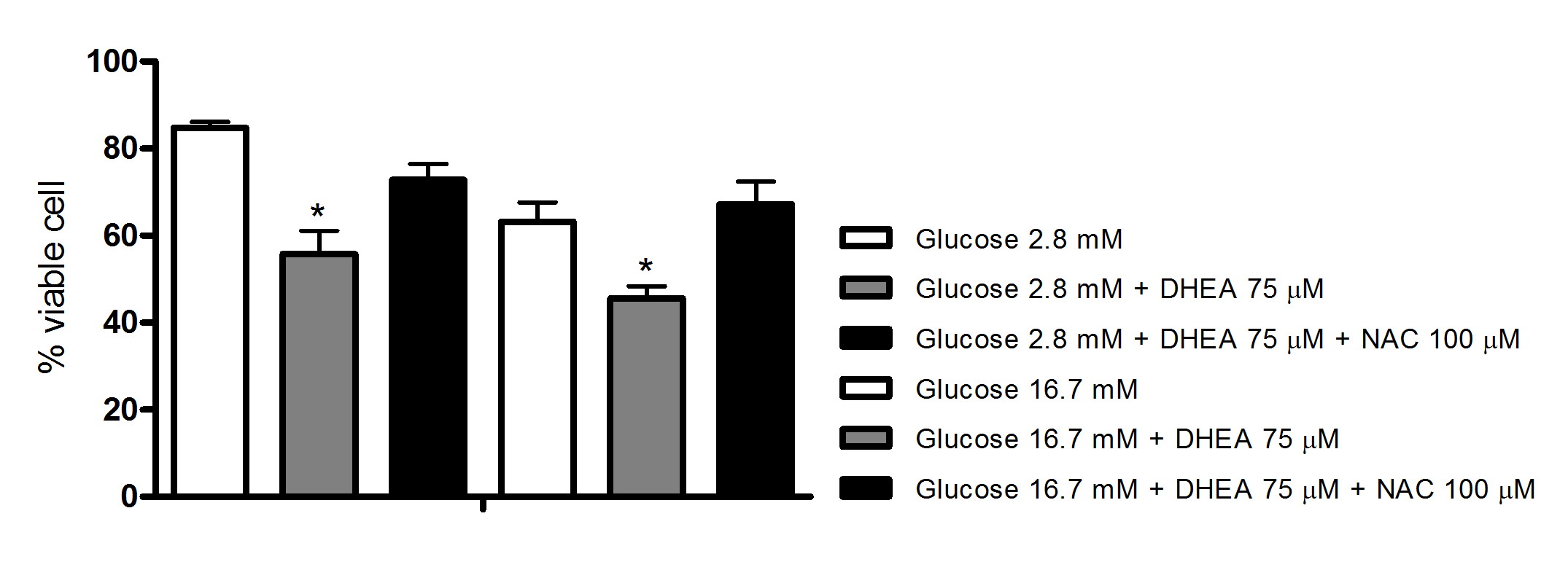

Supplement: S7 Fig — (JPG) [file pone.0158166.s007.jpg]

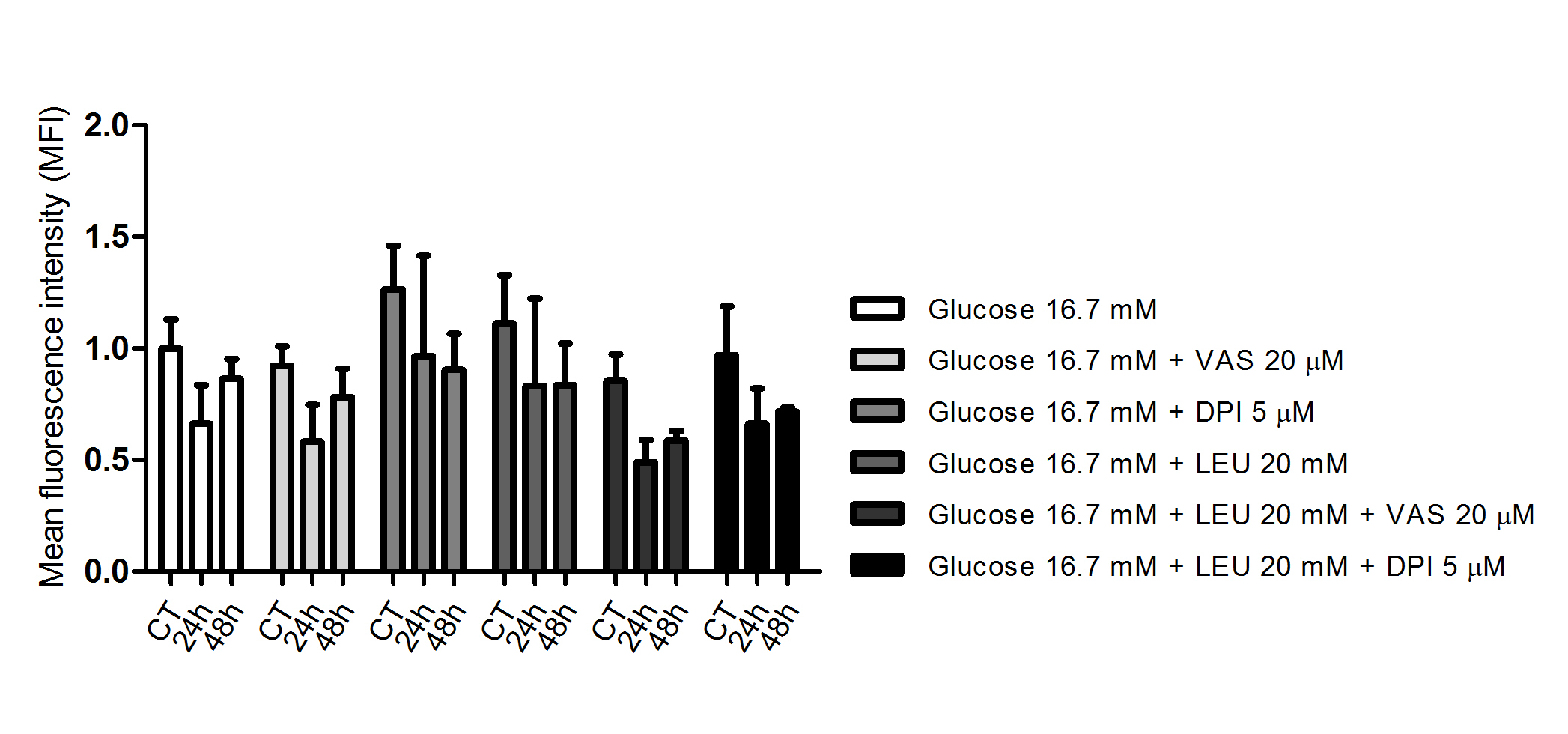

Supplement: S8 Fig — (JPG) [file pone.0158166.s008.jpg]
